# Supplementary material for: Standardization of Semi-Quantitative Dot Blotting Assay—Application in the Diagnosis, Follow-Up, and Relapse of Paracoccidioidomycosis
Source: Microorganisms. 2024 Feb 8;12(2):351. doi: 10.3390/microorganisms12020351 (PMC10892552; doi:10.3390/microorganisms12020351)
Supplement: Supplementary file 1 [file microorganisms-12-00351-s001.zip › microorganisms-2798101-supplementary.pdf]

Supplementary materials

Supplementary table S1. Double agar gel immunodiffusion test performed in 42 patients with active paracoccidioidomycosis, before treatment. Determination of sensitivity and (1 – specificity) of this test as to different dilutions, variables used to determine the cut-off point.

Pacients (n=42)				Controls (n=42)		
Title (cut-off)	Score	Serum + (number)	Sensitivity	Negative serum (number)	Specificity (Spec)	1-Spec
Undiluted sérum	1	31	0.738	42	1.000	0.000
2	2	25	0.595	42	1.000	0.000
4	3	20	0.476	42	1.000	0.000
8	4	16	0.381	42	1.000	0.000
16	5	12	0.286	42	1.000	0.000
32	6	09	0.214	42	1.000	0.000
64	7	06	0.143	42	1.000	0.000
128	8	05	0.119	42	1.000	0.000
256	9	04	0.095	42	1.000	0.000
512	10	03	0.071	42	1.000	0.000
1,024	11	03	0.071	42	1.000	0.000
2,048	12	01	0.024	42	1.000	0.000
4,096	13	00	0.000	42	1.000	0.000

Supplementary table S2. Dot blotting assay performed in 42 patients with active paracoccidioidomycosis, before treatment. Determination of sensitivity and (1 – specificity) of this test as to different dilutions, variables used to determine the cut-off point.

Pacients (n=42)				Controls (n=42)		
Title (cut-off)	Score	Serum + (number)	Sensitivity	Negative serum (number)	Specificity (Spec.)	1-Spec
Undiluted serum	1	33	0.786	42	1.000	0.000
2	2	33	0.786	42	1.000	0.000
4	3	33	0.786	42	1.000	0.000
8	4	33	0.786	42	1.000	0.000
16	5	33	0.786	42	1.000	0.000
32	6	33	0.786	42	1.000	0.000
64	7	33	0.786	42	1.000	0.000

128	8	32	0.762	42	1.000	0.000
256	9	31	0.738	42	1.000	0.000
512	10	30	0.714	42	1.000	0.000
1,024	11	28	0.667	42	1.000	0.000
2,048	12	24	0.571	42	1.000	0.000
4,096	13	22	0.524	42	1.000	0.000
8,192	14	16	0.381	42	1.000	0.000
16,384	15	07	0.167	42	1.000	0.000
32,768	16	05	0.119	42	1.000	0.000
65,536	17	03	0.071	42	1.000	0.000
131,072	18	00	0.000	42	1.000	0.000

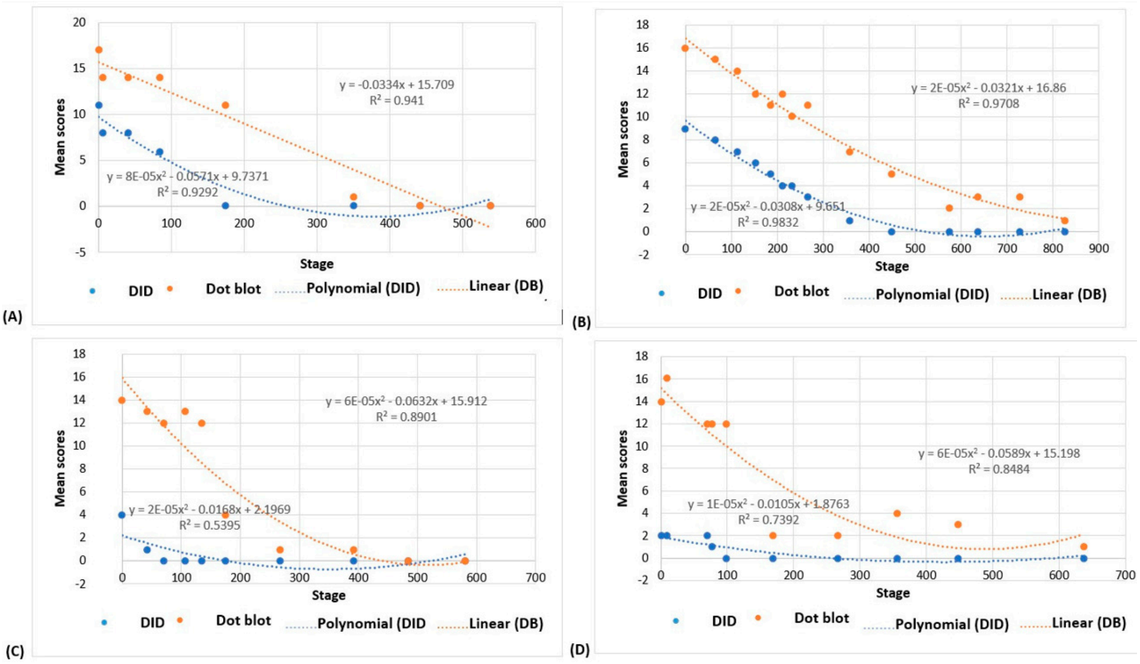

Supplementary Figure S1. Polynomial regression representing the variation of the scores of *anti-P. brasiliensis* antibody serum levels, determined by the dot blot assay and by the double agar gel immunodiffusion test, during the follow-up period. Individual evaluation of four paracoccidioidomycosis patients with the acute/subacute form and their respective severities - A) acute/subacute severe, B) acute/subacute severe, C) acute/subacute moderate, D) acute/subacute moderate.

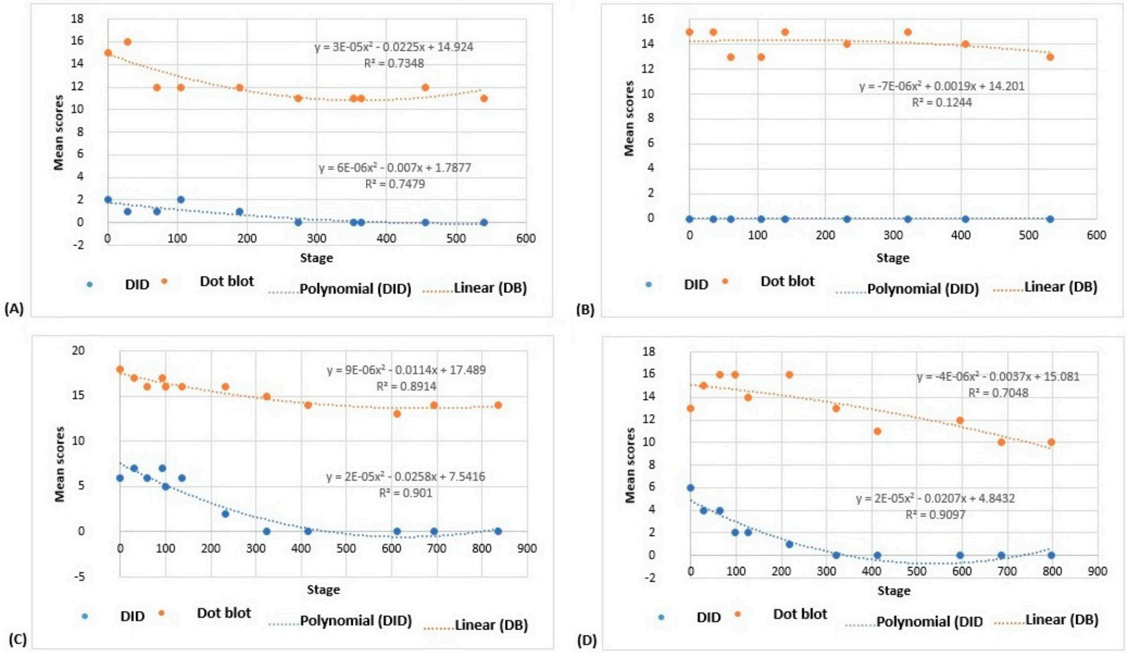

Supplementary Figure S2. Representative polynomial regression showing the variation of the scores of *anti-P. brasiliensis* antibody serum levels, determined by the dot blot assay and by the double agar gel immunodiffusion test, during the follow-up period. Individual evaluation of four paracoccidioidomycosis patients with the chronic form and their respective severities, A) chronic mild, B) chronic mild, C) chronic moderate, D) chronic moderate.
